# Continuous positive airway pressure in managing acute respiratory distress in children in district hospitals: evidence for scale-up

**DOI:** 10.4314/gmj.v55i3.7

**Published:** 2021-09

**Authors:** Frank Baiden, Patrick T Wilson

**Affiliations:** 1 Clinical Research Department, Faculty of Infectious and Tropical Diseases, London School of Hygiene and Tropical Medicine, Keppel Street, London WC1E 7HT, United Kingdom; 2 Department of Paediatrics, Division of Critical Care Medicine, Columbia University Medical Center/New York-Presbyterian Morgan Stanley Children's Hospital, 3959 Broadway, New York, NY 10032, United States of America

**Keywords:** Respiratory distress, CPAP, district hospitals, Ghana, Africa

## Abstract

In children, acute respiratory distress (ARD) is a clinical presentation requiring emergency management, including mechanical ventilation. Mechanical ventilators are lacking in sub-Saharan Africa. Continuous Positive Airway Pressure (CPAP) is an alternative form of non-invasive respiratory support that has been used in high-income countries for over four decades. Its use in sub-Saharan Africa is, however, limited and often restricted to neonates. Controlled trials in Ghana have shown that the use of CPAP in children younger aged 1–12 months reduces 2-week all-cause mortality from ARD by 60% (RR 0·40, 0·19–0·82; p=0·01). The absolute reduction in mortality of 4% implies one infant life saved for every 25 children treated with CPAP. This paper reviews the findings of the trials in Ghana and contrasts the findings with those of trials in Bangladesh and Malawi. It makes the case that implementation research (rather than more controlled trials) is now needed to support the routine, safe and effective use of CPAP in managing ARD in older infants in district hospitals in Ghana.

**Funding:**

None declared

## Introduction

In the emergency management of acute respiratory distress in children, there is a critical need to effectively deliver sufficient oxygen and provide respiratory support to decrease energy expenditure secondary to increased work of breathing. How to do this safely and quickly remains a challenge in emergency paediatric practice. The challenge is even more daunting in district hospitals in sub-Saharan Africa, where options in treatment modalities are often limited.

Acute respiratory distress (ARD) is a clinical presentation that results from a compromise in the proper oxygenation of circulating blood. The clinical manifestations of ARD are often the result of the body's attempt to compensate for reduced blood oxygenation and increased carbon dioxide levels. Relieving ARD through assisted ventilation is an approach that *buys* time to allow for definitive treatment of the underlying disease process. In the absence or delay in effective respiratory management, a rapid, irreversible cascade in metabolic deterioration can lead to death.

Pneumonia, diarrhoea, and severe malaria are common treatable childhood illnesses in sub-Saharan Africa that kill more than 2 million children younger than five years every year.^1^ In the acute phase, these conditions present as undifferentiated acute respiratory distress for which mechanical ventilation is sometimes needed to improve the chances of survival. Most district hospitals in Ghana do not have access to mechanical ventilators, and thus, nasal oxygen is used as an alternative treatment approach^2^,^3^ Using a nasal cannula connected to an oxygen source such as an oxygen cylinder, oxygen concentrator, or walled oxygen are available at most district hospitals. In the absence of a mechanism to maintain a higher than zero positive end-expiratory pressure (PEEP) in the alveoli, nasal oxygen delivery alone may not be sufficient to overcome respiratory failure and may lead to the inefficient use of oxygen.

Continuous Positive Airway Pressure (CPAP) is a form of non-invasive respiratory support that delivers constant PEEP to prevent atelectasis, re-recruit and stabilise collapsed lung alveoli, and widen narrowed airways.^4^,^5^

The positive pressure reduces the work of breathing and enhances oxygenation in children with ARD.^5^,^6^ CPAP has been used in high-income countries for over four decades to manage respiratory distress in neonates and primary pulmonary disease in older infants.^3^,^7^,^8^

Bubble CPAP (bCPAP) is a simplified variation of CPAP where expiratory resistance is delivered via a tube whose distal opening is put underwater. Customised bCPAP devices that run entirely on humidified air/oxygen flow are used in low- and middle-income countries (LMIC). They are often made from non-reusable components and can cost as little as $3. 9,10 Complications that can result from CPAP use include nasal tissue injury, abdominal distension, and pneumothorax.

### Using CPAP in older children

The use of CPAP in managing acute respiratory distress in neonates and older children in high-income countries has been reported for many years.^3^,^7^,^8^ Although relatively recent, the extension of such use in neonates in LMICs is currently increasing. However, its use in older children in LMICs is less widely accepted and is mainly limited to clinical trials. The conduct of the trials in district hospitals has also assessed operational feasibility.

In a trial at a district hospital in Dhaka, Bangladesh, Christi et al. compared three modalities for delivering oxygen to children younger than five years who presented with severe pneumonia and hypoxaemia. The trial enrolled 146 out of the planned target of 640 children. Treatment failures (clinical failure, intubation and mechanical ventilation, death, or termination of hospital stay against medical advice) were recorded in 6% of children who received CPAP, 24% of children who received lowflow oxygen therapy and 13% of children who received high-flow oxygen therapy. The proportion of deaths among children put on CPAP versus those put on low flow oxygen was 4% to 15%. The trial was stopped early on account of clear benefits with the use of CPAP. Reanalysis of the data using Bayesian predictive modelling showed that the probability that CPAP was superior to high flow nasal cannula (the third arm) was 0.98, 0.98and 0.72 for three possible scenarios that were explored. 10–12

In Ghana, an initial proof-of-principle randomised-controlled trial showed that CPAP decreased respiratory rate in children aged 3 to 59 months old who presented with respiratory distress. The trial showed that in some rural and semi-rural settings, CPAP could be successfully used by physician-supervised nurses to manage acute respiratory distress.^13^ The mean respiratory rate of children who received immediate CPAP fell by 16 breaths/min (95% CI 10–21) in the first hour compared with no change in children who had CPAP delayed by 1 hour (95% CI -2 to +5). The study was stopped after enrolling 70 out of 96 subjects because the predetermined endpoint had been reached.

In the second trial in Ghana, 2200 children aged 1 to 59 months presenting with undifferentiated acute respiratory distress were enrolled in two semi-rural district hospitals. This was an open-label, cluster, crossover trial. In unadjusted analysis, CPAP was shown to reduce 2-week allcause mortality by 60% in children younger than 1 year (RR 0·40, 0·19–0·82; p=0·01). The absolute reduction in mortality in children under one year of age was 4%, meaning one infant life saved for every 25 children treated with CPAP. The study found that after adjusting for study site, time, and clinically important variables, the odds ratio (OR) for 2-week mortality was 0·4 in children aged under six months, 0·5 for children aged 6–12 months, 0·7 for children aged 12–24 months, with no benefit in those 24 months or greater (OR 1.0). This study also demonstrated that physician-supervised nurses could successfully use CPAP in semi-rural district hospitals.

In an open-label, randomised, controlled trial in a district hospital in Malawi, McCollum and colleagues enrolled children aged 1 to 59 months who presented with WHO-defined severe pneumonia and either HIV infection or exposure, severe malnutrition, or oxygen saturation of less than 90%.^2^ The trial found higher mortality among children who received CPAP compared to children who received low-flow nasal cannula oxygen (17% versus 11%; relative risk 1·52; 95% CI 1·02–2·27; p=0·036). Suspected adverse events related to treatment occurred in 3% of children who received CPAP compared with <1% in children who received oxygen. The trial was stopped for futility after 644 of the planned 900 participants had been enrolled.

In summary, four trials have explored the use of CPAP in older children (1–56 months) in district hospitals in three LMICs. While the larger trial in Ghana went to completion, those in Bangladesh and Malawi were stopped because of the early emergence of clear outcomes. An editorial that accompanied the publication of the trial in Malawi explored possible reasons its outcome was so different from the trials in Bangladesh and Ghana.^14^ Very important differences were highlighted. Whereas the trials in Ghana and Bangladesh excluded children who had conditions for which the use of CPAP was unlikely to be beneficial (e.g. abnormal Blantyre Coma Score, hypotension, impending respiratory failure, asthma, upper-airway obstruction, persistent emesis), the trial in Malawi did not. Enrolment into the trial in Malawi was initially limited to children who had pneumonia and HIV (or had been exposed to HIV) or severe malnutrition.

Additionally, while the children enrolled in the trials in Ghana and Bangladesh were managed by physician-supervised nurses, those enrolled in the trial in Malawi were managed by clinicians and nurses who worked “without daily physician supervision”.^14^ It is evident that although the trial was reportedly designed to reflect “real world” district hospital settings in Malawi, it enrolled high-risk children who needed care beyond what CPAP is physiomechanically designed to do.^10^

CPAP has been shown to improve the management of children who have moderate-to-severe respiratory-related conditions. However, there are important contraindications to its use that need to be adhered to regardless of the setting in which it is used. Training nurses to make this distinction and ensuring daily physician supervision is a health system challenge that varies across different settings in SSA. These challenges are amenable to improvement, as demonstrated in the trials in Ghana and other studies in Malawi.^7^,^15^,^16^ It is also relevant to point out that Malawi has the most advanced rollout of neonatal CPAP in sub-Saharan Africa, outside of South Africa. Neonatal CPAP has been introduced into all district hospitals. The use of CPAP in neonatal presents a much more delicate consideration than use in older infants. Ironically, Malawi has rolled out neonatal CPAP across district hospitals while use in older infants is withheld.

The current academic debate regarding the sufficiency of the evidence to support the scaling up of CPAP in district hospitals in LMICs has interesting parallels in the debate that preceded routine use of CPAP in high-income countries in the early 1970s. At a point in time, it was felt that it was inappropriate to continue with more of such trials in the face of evidence from both noncontrolled and controlled studies.^17^,^18^ In recent times and related to CPAP use in LMICs, Jansen et al. (2014) have opined that if CPAP remained an experimental therapy in these countries, needless morbidity and mortality would result. (Jensen et al. 2014) Ekhaguere *et al.* (2019) further add that high cost randomised controlled trials on CPAP use in LMICs are no longer needed. What is needed are high quality operational research on how CPAP can be safely and sustainably used in district hospitals in LMICs.^10^

District hospitals are the first level referral facilities within the health system in Ghana. The number of beds in a district hospital is typically between 50 and 60. Every hospital is required to have a doctor(s), nurses and other medical staff. In 2017, the Ghana Health Service reported that 128 out of the 137 district hospitals had at least one doctor. District hospitals are required to have the capacity to undertake emergency and elective surgeries. They are therefore equipped to administer oxygen and resuscitate acutely ill patients, including children.^19^

A survey in 2005 reported that 90% of district hospitals in Ghana could deliver oxygen as part of care.^20^

In most parts of Ghana, unless a child who presents with acute respiratory distress at a district hospital is well-resuscitated, there is a high probability that the child will die before reaching a regional referral hospital. Apart from the trials referred to earlier, very little is known about CPAP use in district hospitals.

### Sustainability

The first reported randomised controlled trial of CPAP use in Ghana (proof-of-principle study referred to above) took place in the district hospitals in Kintampo, Mampong, Nkoranza and Wenchi between June and November, 2011.^16^ Sixteen months after the end of the trial, the investigators undertook as evaluation of the extent to which the skills and equipment necessary for CPAP use have been maintained. They found that nurses who were trained in formal 4-hour sessions at the beginning of the trial retained good skills and knowledge. They performed better on assessment than nurses who had been trained through informal approaches. Seven of eight CPAP machines that had been donated to the facilities after the trial were functional. However, five of eight oxygen concentrators and three of four electric generators that had been procured to facilitate the trial were non-functional.

The second trial took place in the district hospitals in Kintampo and Mampong.^3^ Given the clear evidence from the earlier proof-of-principle trial, the investigators in the second trial did not consider it appropriate to implement a randomised design. They instead opted for a cluster crossover design.^3^ At the end of the trial, the CPAP machines, oxygen concentrators and generators were, upon request, donated to the two hospitals. In an unpublished assessment of the functionality of the equipment six years after the completion of the trial in 2015, we found that all eight CPAP machines and two oxygen generators were functional and still in use.

There has also been substantial improvement in oxygen and power supply in all four district hospitals that have been involved in the CPAP trials between 2011 and 2015. The continuous routine use of CPAP in these facilities does not require the special provision of oxygen concentrators and power supply. While all the staff trained in Kintampo hospital were still at post, four out of the five staff trained at the Mampong Hospital had been transferred to other health facilities.

Lessons learned from the implementation of CPAP in other sub-Saharan countries also guide Ghana. In Kenya, CPAP was successfully introduced and sustained in ten government hospitals using a two-day training-of-trainers curriculum.^21^ The CPAP training curriculum has been transferred and incorporated into the continuous professional training program of the Kenya Paediatric Association. The association continues to train healthcare providers throughout the country with the assistance of a local non-governmental organisation. Through this program, prospective observational CPAP usage and safety data have been collected and used to develop national neonatal CPAP guidelines. A similar program was implemented in Rwanda, but follow-up data is not available.

### Way forward

The evidence on CPAP use in managing acute respiratory distress in children in district hospitals in Ghana is positive. Efficacy has been established in its use in children aged less than one year, and there is evidence that health workers in district hospitals can be trained to use the device safely. To move the evidence into routine practise, however, clearly foreseeable challenges will need to be addressed. These include developing model facilities of excellence, formalisation of training and incorporation into standard treatment guidelines. As suggested by Ekhaguere *et al.* (2019), this will require implementation research designed to address operational challenges and facilitate learning-by-doing and experience-sharing across facilities and among health workers. The lack of mechanical ventilators in most health facilities in Ghana (including district hospitals) and shortage of physicians highlight the need for further capacity development and brings CPAP's potential to the forefront. Surmountable health system challenges are no justification for withholding CPAP to manage acute respiratory distress in infants in district hospitals in Ghana based on the currently available data.
